# Supplementary material for: TcJAV3–TcWRKY26 Cascade Is a Missing Link in the Jasmonate-Activated Expression of Taxol Biosynthesis Gene DBAT in Taxus chinensis
Source: Int J Mol Sci. 2022 Oct 29;23(21):13194. doi: 10.3390/ijms232113194 (PMC9656678; doi:10.3390/ijms232113194)
Supplement: Supplementary file 1 [file ijms-23-13194-s001.zip › Figure S2.pdf]

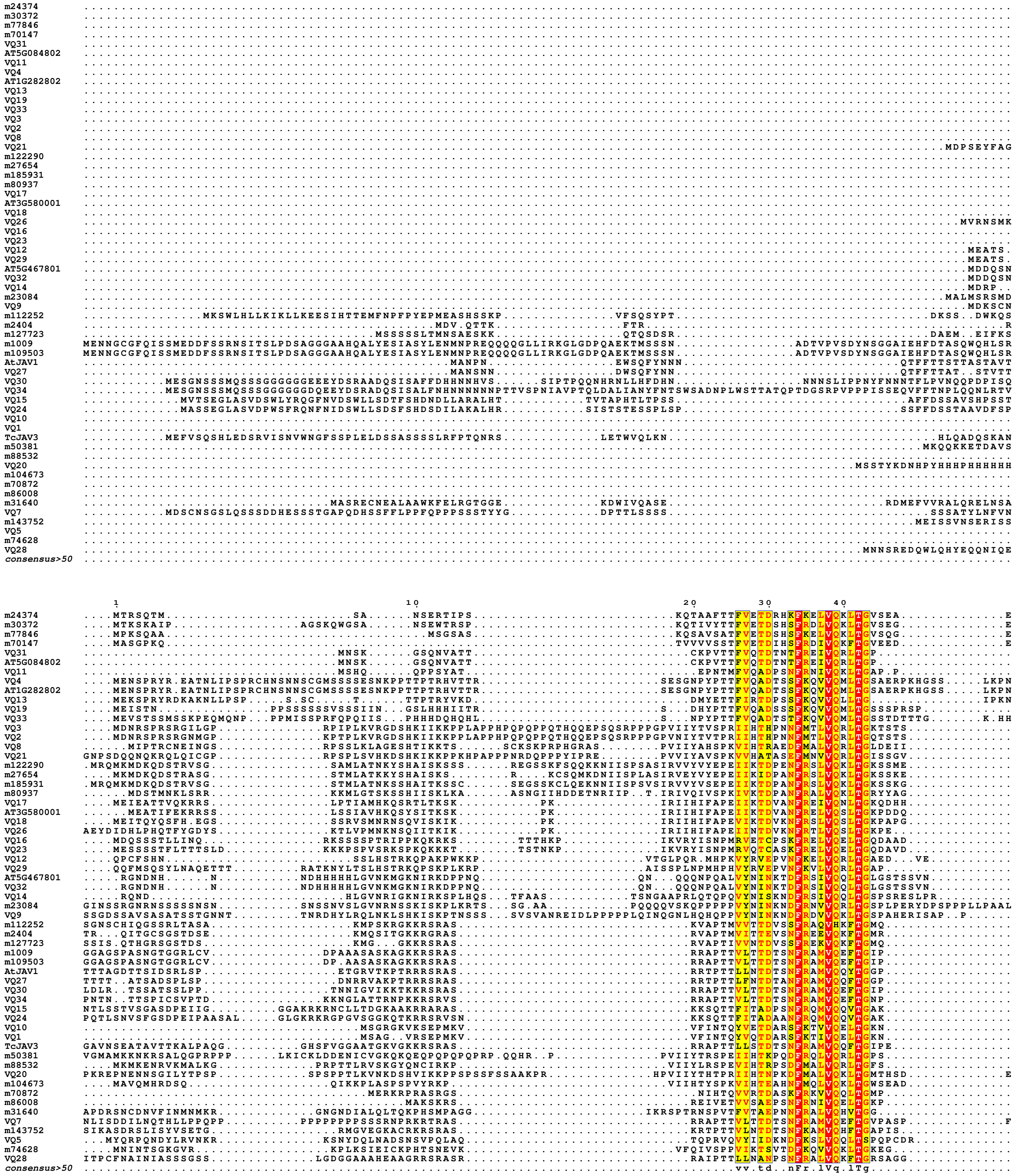

m24374
m30372
m77846
m70147
VQ31
AT5G084802
VQ11
VQ4
AT1G282802
VQ13
VQ19
VQ33
VQ3
VQ2
VQ8
VQ21
m122290
m27654
m185931
m80937
VQ17
AT3G580001
VQ18
VQ26
VQ16
VQ23
VQ12
VQ29
AT5G467801
VQ32
VQ14
m23084
VQ9
m112252
m2404
m127723
m1009
m109503
AtJAV1
VQ27
VQ30
VQ34
VQ15
VQ24
VQ10
VQ1
TcJAV3
m50381
m88532
VQ20
m104673
m70872
m86008
m31640
VQ7
m143752
VQ5
m74628
VQ28
consensus>50
1
10
20
30
40
vv.td..nFr.lVq.lTg

```
                50        60                   70       80                          90      100
m24374          KLCVTTPARSSGN..........AGTKPEGNHSPVIKSVIEVGVQKSGLKL...................HQRRQFQRKVPAP.........................................
m30372          KLPVTINGRNSVKGS......VEAGIKSEGDCSALIKSGIEVGTQKSAFNL...................HQRRKFQKRLEIKPGF.................YHDSQNETTILPGGKKY....
m77846          KIPVTMPSRISQKG........CAEIKIDGNYCAVMKIGMEVGPQKSEFKL...................HKRRQIKLGLTDLD.......................PDDKKIMLPGNNY....
m70147          KLPVTMPERNSKKGS......AAAGMRSNG......KSGIELGPQKTEFKL...................HKRNQAQRKLEINMG...................LTESHNTVISRGNNNF....
VQ31            .TENNAAAATPEATV......IKTAIQ........KRPTSKLHERRQCMRP...................KLEIVKPPLSFKPT........................................
AT5G084802      .TENNAAAATPEATV......IKTAIQ........KRPTSKLHERRQCMRP...................KLEIVKPPLSFKPTG...........................TTPSSKSG....
VQ11            DISSSSFSAVSAAHQ......KLPLTP........KKPAFKLHERRQSSK....................KMELKVNNITNPNDA...........................FSHFHRG.....
VQ4             PTHHQPDPRSTPSSF......SIPPIKAVPNK...KQSSSSASGFRLYE.....................RRNSMKNLKINPLNP..........................VFNPVNSAFSP..
AT1G282802      PTHHQPDPRSTPSSF......SIPPIKAVPNK...KQSSSSASGFRLYE.....................RRNSMKNLKINPLNP..........................VFNPVNSAFSP..
VQ13            PT.HQPDPR.FPPFH......SIPPIKAVTNK...KQSSS....FRLSE.....................RRNSMK....HYLN............................INPTHSGP....
VQ19            DSPRPPTTPSGKGNF......VIPPIKTAQPK...KHSGNKLYERRSHGGF...................NNNLKNSLMINTL...............MIGGGGAG...SPRFSPRNQE.....
VQ33            EAPSPVNNNNKGSSF......SIPPIK........KTNSFKLYERRQNN.N...................NMFAKNDLMINTLRLQNS.......QRLMFTGGNSSHHQSPRFSPRNSSSS...
VQ3             .TTSSSYSSSTSAPKDASTMVDTSHGLI....SPAARFAVTEKANISNELG...................TFVGGEGTMDQYYHYHHH...................HHHQEQQHQNQGFERPS
VQ2             .TTSSSSSSSTSEPKDTSTMVDTSHGLI....SPAARFAVTEKANISNELG...................TFVGGEGTMDQYYH.......................HHHQEQPHQNRGFERPS
VQ8             .RRNTSE.........................SSSSVVTEEVNVGDDNTAA...................PFSQDRTQRQK...........................................
VQ21            .FLESGGG................GDV.....SPAARLASTENASPRGGKE...................PAARDETVEINTAM..........................EEAAEFGG......
m122290         .NEEINSTKR.............SSCT.....ETCEENPEKPCFVGLSQGF...................NN..LGMLF.............................PGLMNHESVNE.....
m27654          .N..........................................................................................................................
m185931         .E..........................................................................................................................
m80937          .SSKKPIQKK.............PIPQ.....VSPEQLDSIDFKRSVGQDL...................SP..ADHFHGSAVVDDHFFWKNSAQNEFVWCEDQVVSPPAEINTEYLEED....
VQ17            .HHDLPHQKG.............LKRN.....PRSRRSHDHHEVHDMNKS.........................HGFCINSDEEE......................EGMVSMTWGN.....G
AT3G580001      .RTSKAKPR..............RDMH.....RLHRQVQDMINTEKLREPE...................HC..DQEFCLN.AEE..........................LSMTWNGNNG..A
VQ18            .EAKTGKKRA.............KSRI.....TTPQEPVCDDHQPVNRLS..........................GFTGLLANGG......................NHQVKEEWGSG.....
VQ26            .ITKTGSKKK.............ITNT.....NFPAPPGLPQDSGQEHTA..........................ELDNRII..G......................SHVVKEEWGS......
VQ16            .LPPSPTTFT.............AVDL.....HRPCESEMNLEP..LDGEV...................RG....EYYSPLD.........................EEVFNAPQMSAG....
VQ23            .LQPEPIYSP.............SSDD.....HNLSPPAENLAPRVLHQEP...................FGERDSDCYEPLNA........................EDMFLPDQMSAG....
VQ12            ..........Q..............EV.....HQVEAKPVKISDDTTT........................................................................A...
VQ29            .........................EH.....ETVQAKPLKTSDDAAK........................................................................Q...
AT5G467801      .PPQTNHPKPP............NSRL.....VKVRPAPLTQLNHPPPPPP...................PPPPVQSVP.IASEP..........VQPVN......QFSSNPAESPISAYMRYL
VQ32            .PPQTNHPKPP............NSRL.....VKVRPAPLTQLNHPPPPPP...................PPPPVQSVP.IASEP..........VQPVN......QFSSNPAESPISAYMRYL
VQ14            .PPQNNSLRPQ............NTRL.....QRIRPSPLTQLNRPAVPLP...................SMAPPQSHPQFARQPPHQPPFPQTTQQPMMGH..RDQFWSNTAESPVSEYMRYL
m23084          PVTPAPNPKPP............NSRL.....HKIRPPPLSALGSAAPPPM...................QLRSPQFMN.FSPLPP.........LTPGD....AVWAGNIAMESPVTRYMKYL
VQ9             .QQPIHHPKPQQ...........SSRL.....HRIRPPPLVHVINRPPGLL...................NDALIPQGSHHMNQNWTGVGFNLRPTAPLSPLP.PLPPVHAAAESPVSSYMRYL
m112252         AQPTTNS.............................DPSAAKSAIPFP........................FKPMPKCPLDQ..........................NLVGPFSYHSYNTTN
m2404           PPPTKNY.............................DPSAANSSITLP........................FMPMPKRFLDQ..........................TFEGFSNYHSYNTTD
m127723         TRPATYS.............................DQMAANPTMGIPL.......................IKPMPKRPLDQ..........................TLAGTSSYHNYSTIN
m1009           PAPFASA.................SPF.....PRARFDFLGAPRSLAN.P....................YLLRPFPQRMAAGTHSFA.......PS......SAPTLQSLPQAPLFQSLLHPG
m109503         PAPFASA.................SPF.....PRARFDFLGAPRSLAN.P....................YLLRPFPQRMAAGTHSFA.......PS......SAPTLQSLPQAPLFQSLLHPG
AtJAV1          SAMAFGSG................NTT.....SAFSLTSSSDPSAGSSQQ.......................APWQY.NFQ.............................PHAPLQPPQRP..
VQ27            SAVAFGS.................SPS.....SGFSLTSS.DPTAGVSS........................SPWQYANLQN..........................QMAHNELMQQQRP..
VQ30            APPLFNNN................SIV.....NTTRLNTFLGLSSSSP.........................NTYNTNNLLLRPFAQ.......KL......TP..ATPLLSGSQIQQYQ...
VQ34            STPFTGLS................SSF.....PRSRFDLFGSSSSSSSRPMKPFPHKLISPSTLNHHYLPPSSEYHHHHQHQNLLLN........MN......TPNISNPFLNNSLTDQPKPSS
VQ15            YIDDSSSF................GIF.....DPIVKPEPLRFVNKLP........................CGPSDRSTAVP..........................MLDTSAFLSNHH...
VQ24            FLGSSNS..................IF.....APIVKPEPHRLASRLPPS......................CGNLDRSSAVP..........................TLDTSSFLSNHH...
VQ10            AIVAAGPF................DS.........PSAFDGRCYDGGS............................KI....G............................EDTRQLHGGG...
VQ1             AVVADGPY................E.............FSGQGYGGK................................................................DSSQRFCGVG...
TcJAV3          .SPYASQ............................GRSLYRFTPPQHN..............................................................YYSSFLFQDR....
m50381          .KNKKKKR................PKKK....AAPQGSSSQVPQTTLKPP.....................CEFNNAAAAAAAYP..........................LPPLPPSSSTQS.
m88532          .SSSSSAH................LMED....SKTVKTSPSVFNHVAHDD....................DNYDRLELQREEKS........................YQIIPPPSVCRN....
VQ20            DLGGGNAMTDPG...........VVKSIN...RTVSEPTVDRKNNRNRCGGGN.................NYLRNYSGAGNGKGIFFN....................NTMISEDSESSSVITT
m104673         KKRKRQKQKQKMKK.........RSEESLQGMMPDPTPCLNIAAASAKMVMN..................KCKEISSLRTDSTP.....................AHFMPDMSSYGCAGAVEEV
m70872          .KPEKP..............................LQLVLKPVARRP...............................................................IVEQISPD.....
m86008          .KPKLET.................IHES....PSLLKPVAKRPLVDREQ.....................LCAKTCPWISSLS..........................SSSSSSSSSSAAHK.
m31640          .VDDTAMRG.......................AEVLKPRPKRPCTSSRVLN......................DSVPFLSHSR............................KPSFSVFPAALP.
VQ7             SHPFSSTTRR.............FDIFRSPSDPLTYNPFRPIPQKPLNPSTS..................SLLNLHHHTTTSMTFP.....................DLPLPQTHQVSTFQSLL
m143752         .NPNTFAS...........................SKPGMGGYHNDFFP.........................PNKFMAAEN...........................SIYAPAFN......
VQ5             LPQNIPKHQDIR.............PEPIN..WTSSIPPSAMAVQEDPDVS..................LYMAYLQSLLEESSGS.......................NGDQFEEPFDKYHSHM
m74628          .SSSIQS............................HKNVIPVPLELPN...............................................................YGQPQNSN.....
VQ28            .ESNRRKGP..............VTLDFGSPTTISKEAIFPVSSDRSNNN....................LDHVVNQHVSNEQR.......................HVTWSGTESMMSYQLGE
consensus>50    ............................................................................................................................

                            110                                                                                       120
m24374          .......DPTLIPSPITPLG................................................................................PADPFTCYNN.............C
m30372          .....VISPTVIPSPITPLG................................................................................PVDPFNTSP...............
m77846          .....MLAATLIPSPVTPL.................................................................................EANPFSCYS..............Y
m70147          .....VLSPTLIHNRITPLE................................................................................HEDPCTASSN.............Y
VQ31            ............................................................................................................................
AT5G084802      .......NTNLLTSPVGTP...................................................................................SSLFSNLS..............
VQ11            .........FLVSPVSHL....................................................................................DPFWARVS..............
VQ4             .RKPEILSPSILDFPSLVL...................................................................................SPVTPLIP..............
AT1G282802      .RKPEILSPSILDFPSLVL...................................................................................SPVTPLIP..............
VQ13            ...PEILTPTILNFPALDL...................................................................................SPDTPLMS..............
VQ19            .....ILSPSCLDFPKLAL..................................................................................NSPVTPLKQG.T......NGNEG
VQ33            ..ENILLSPSMLDFPKLGL..................................................................................NSPVTPLRS...........ND.
VQ3             FHHAGILSPGPNSLPSVSP....................................................................................DFFST................
VQ2             FHHAGILSPGPYSLPSVSP....................................................................................DFFST................
VQ8             ......LTDMPLFTPSSMT.........................................................................................................
VQ21            YAPG.ILSPSPALLPTAST....................................................................................GIFSP................
m122290         ....VPLLKNNSPFCNMCD....................................................................................QSGVY................
m27654          ............................................................................................................................
m185931         ............................................................................................................................
m80937          ...AVKMRDNFSGFFGGFE....................................................................................EQDIF................
VQ17            DESSGGFLNGLGDLDGFIQ....................................................................................ELGEF................
AT3G580001      GESSGGFLNGLGDFEGFIQ....................................................................................ELGEF................
VQ18            .DQSTSNTNTYFDLEGLIQ....................................................................................DVGED................
VQ26            ....SSNTNTYFDFDGLI.....................................................................................DLDED................
VQ16            ..LSGFFSSGFYNVNALG.....................................................................................SIGSL................
VQ23            ..FSGFFSNGFYNVNDFG.....................................................................................SIDSM................
VQ12            .......KDNPFAFDL...........................................................................................S................
VQ29            .......SSSSFAFD.............................................................................................................
AT5G467801      IE....SSPVGNRVQP..............................................QNQN..........................PVQPS.....TGLFQS................
VQ32            IE....SSPVGNRVQP..............................................QNQN..........................PVQPS.....TGLFQS................
VQ14            QSSLGDSGPNANQMQPGHEQRPYIPGHEQRPYVPGNEQQPYMPGNEQRPYIPGHEQRSYMPAQSQSQSQPQPQPQPQQHMMPGPQPRMNMQGPLQPNQYLPPPGLVPSPVPHNLPSPRFNAPVP
m23084          QDSVAGGGGGGGGFGGG..................G.........................AYNPSPR......................AMQLQHR...PAAPLLPS................
VQ9             QNSMFAIDSNRKEFSG.............................................LSPLAP.........................LVSPRWYQQQENAPPSQHN...........SF
m112252         ..SLSSFSN.SATQKFP..........................................................................................................F
m2404           ..SLSSFSS.SDIHKFP..........................................................................................................F
m127723         ..MLSSFTNNSGASNFPS.........................................................................................................N
m1009           GPNLVHKTTTNISEAEAEAEGCRRVRL..................................................................LATLEEGRCGALASPRLR............L
m109503         GPNLVNKTTTNISEAEAQAQAQAHMK...................................................................AGLLEELGLNTHALPDLIPW.HAGQDNSAAC
AtJAV1          ....YMFSLNNVNPVVG..........................................................................................................Y
VQ27            ....YMFSSSNNVSTLS..........................................................................................................Y
VQ30            .NPNNGFEDMNLQALLQ.....................................................................................AHISNPRSNE..HDQFGLGMMQ
VQ34            LRTSNGFGHVDVGTNFEGLHNIIVSS...................................................................SSSMTQPTLNTIHGPEKST..EAENDNNMLR
VQ15            .....QEN...LAVGNA..........................................................................................................F
VQ24            .....QEN...IITDLG..........................................................................................................A
VQ10            ............GGGGR..........................................................................................................M
VQ1             ............KEAER..........................................................................................................G
TcJAV3          ......MNTPPFFLPR............................................................................................................
m50381          .QLYSGLLSPFPISPLP......................................................................................PFSPN................
m88532          ....DYPSEVISPVQWS...........................................................................................................
VQ20            EENIGEHGQVNSSLPYSAVAIP.................................................................................PQPPP...............H
m104673         MPSYFLFNNNAPNMASSDQ.........................................................................................................
m70872          .......NRVFSSLTTTS..........................................................................................................
m86008          .STPQSTSSLPQSILVE...........................................................................................................
m31640          ....SVFNGSSPALHNP...........................................................................................................
VQ7             SHQHHHQPTLSSLHDLDSMN.................................................................................LGALQQN................
m143752         .....VAKSSSLAYDFAP..........................................................................................................
VQ5             MAQSQPQDPTQSMPQSNG...................................................................................FEPFPSS................
m74628          .....AFVHDEVHYQVP...........................................................................................................
VQ28            ETNSAVYSSGYDDQDLPR..........................................................................................................
consensus>50    ............................................................................................................................
```

```
                 130                                        140        150            160
m24374        SPSCSSTPPS..................................CNQSPYFG.EEEGFCF.....QASPIRVKREPELLPLF..........................................
m30372        SYCYSSTPTS..................................CNPSSYLE.EGEKFCF.....QDSAISMKREPELLPLFPLH...............SPTQPNTSFQ..............
m77846        NPSCCSTPSSSS................................CNQSSYLEGEADEFS..........C.LKSEPKLLALFPLH...............PATQPTGI................
m70147        CYNFASASVS..................................CSQSSCLE...EEFGF.....QPSPISLKAKPQLLPLFPMH...............SLTQPTNP................
VQ31          ..EGEPDSCTTNIE...............................EEEKAIKERRFYLHP.....SPRSKPGYTEPELLTLFPLT...............SPNSSGKP................
AT5G084802    LIEGEPDSCTTNIE...............................EEEKAIKERRFYLHP.....SPRSKPGYTEPELLTLFPLT...............SPNSSGKP................
VQ11          PHSAREEHHAQPDK...............................EEQKAIAEKGFYFLP.....SPRSGS.EPAPELLPLFPLR...............SPNGTNHRIHEDNHRDS.......
VQ4           DPFDRSGSSNQSPNE............................LAAEEKAMKERGFYLHP.....SPATTPMDPEPRLLPLFPVT...............SPRVSGSSSASTS...........
AT1G282802    DPFDRSGSSNQSPNE............................LAAEEKAMKERGFYLHP.....SPATTPMDPEPRLLPLFPVT...............SPRVSVNKR...............
VQ13          DPFYRPGSFSQSPSDSKPSF.......................DDDQERSIKEKGFYLRP.....SPSTTPRDTEPRLLSLFPMT...............PIHSPAPSPHDH............
VQ19          DPFDKMSPLS...................................EEERGIADKGYYLHR.....SPISTPRDSEPQLLPLFPVT...............SPRLSPEM................
VQ33          DPFNKSSPLSLGNSS..............................EEDKAIADKGFYLHP.....SPVSTPRDSQPLLLPLFPVA...............SPARNS..................
VQ3           ..IGPTDPQ...........................................GFSSFFN......DFN...SILQSSPSKIQSPS..................SMDLFN........NF.....
VQ2           ..IGPTDPQEGTPYLIPSGDMHSYLSQDEDRGIPHSVKDTRSIGSAYDRYLQSMQTFFVPSEEAGPFNGVGMVRQGGSNMMPGPS..................MGELMAGCGGSLPSDFRPNGR
VQ8           ...............................................LFGSPTQLMYMS......PNR....TDSFRP.LVFKSE.......................................
VQ21          ..MYHQGGM.................................FSPAIPLGLFSPAGFMS......PFR....SPGFTS.LVASPT..................FADFFS...............
m122290       ..SLAEASH.......................................Y...........................................................................
m27654        ............................................................................................................................
m185931       ............................................................................................................................
m80937        ..NTVMESQ.......................................LILPEISFT............NPGGFSQYRPGGFSHF..................Y....................
VQ17          ..PYLPFT........................................IDPAVAS.................SSHLHG.NVFAEP..................HHYA.................
AT3G580001    ..PYLPLSS.......................................IDASASSN.............SSSSSHLHGGSVFSD....................QFA.................
VQ18          ..YFSSFP........................................MRSSSSSQVEGFIFNNNNNNNNNNNNNNNNNTNFDTK..................AHNSS................
VQ26          .........................................................................DNIFSSRWFDLQ..................QQ...................
VQ16          ............................................................................................................................
VQ23          ............................................................................................................................
VQ12          ....PSSSR..............................................F...................WEAFPLLSP....................ANLS................
VQ29          ....PSSS...................................................................WGDFSFQNP....................ANIS................
AT5G467801    .HQTGPNPMS......................................FQSPASQFAL......SPQ.......PRSPFPLFSP..........NFAFS....PRFLG.GSNES....LPPPSP
VQ32          .HQTGPNPMS......................................FQSPASQFAL......SPQ.......PRSPFPLFSP..........NFAFS....PRFLG.GSNES....LPPPSP
VQ14          VTPTQPSPMFSQMY..................GGFP....SPRYNGFGPLQSPTSQFLQP....SPTGYPNMFSPRSPYPLLSPGVQYPQPLTPNFSFSQIAQQGSLGPGAGPSQGPPQPPPSP
m23084        .PRRPPPPLP......................................LPSPTSQYLLP.....SPLP.....SPGSFLSFPSPG.......QFPLNFFTQSPSGQFGFPGGGP....LLPPSP
VQ9           PPPHPPPPSS......................................AVSQTVPTSIP.....APPLFGCSSSPKSPYGLLSP..........SILLS..PSSGQLG...........FPVSP
m112252       IGSAISFPNFSGQP..................................LFSQEQM..LP.......YILSSTDPFINSITAETNNCNSR...................................
m2404         IGSAIS......................................................................................................................
m127723       LGSAISLPNLSTFQP................................DLFEQEQQQNLS.......TAPLALHPFFNSITSHANNAGFL...................................
m1009         RVSIKHGPSIQPDP..................................QIEPAGYSR...................SKIENYNLT.......................................
m109503       KTSYSKSAAAPGDPGRSRNL...........................NLDEPIANSSDS.......HHGRPDRMVDSWISSSDQTLDKLVQ.................................
AtJAV1        SNMNNPNTMVSGVFG.................................TVDGSGGGG.S.......APSSKEATNSNSSSSRLQ........................................
VQ27          PNAVATDGFVGAEES.................................REGGGGGGGGY.......APSSERNSNTTYSEEKTVYYS.....................................
VQ30          TPSTNPPTTTAAANG.................................NINTGDNGSYG.......GSDHEHNNDGTWLSSSSDQRT.....................................
VQ34          SINGNDQPMVQRSDG.................................YTQPGASGSEN.......NLSAVRSEGMVELSWISSSD......................................
VQ15          SGNSSSVGLPSGKP..................................SATADPGGS.........AVEFDNYPTFPTLESWKVM.......................................
VQ24          PTGSFHHQSSAGTT..................................TANVGGGSS.........AVELDSYPSFPTLESWKVM.......................................
VQ10          GTTTEFDRLFKEMP...........................................................HMEELYKLWSEY.......................................
VQ1           VETTEFDSFFREMP...........................................................PVGELYNLWSDH.......................................
TcJAV3        ...VPPPPLN.......................................PSAAEDFGLN........PVFSGPLMLPHLGISDSLIK.....................................
m50381        FFTMSPPGVN......................................TRFHMDLLFSFSP...SPLPPFDQIFPYPLDPFNNVP.......................................
m88532        ..SFPAEEIDLD....................................MFFDLLSSRYSSSQAVLQDFPFMETELQTFNCVYDVDCL.....................................
VQ20          PPPPPPPPSMYDAAGINYG.....................AYLPTFPIFPPANPADNFLCGNQPFANFDDPLFFAPNMRSSFSSSS...............SSGFDGLTEFRDF..........
m104673       ..RQPSNYDPYNQQ..................................RLCFPHNINLPPS...STPPIGEYNFSDFLPKIFAHNVDF....................................
m70872        .CFQTPNPS.......................................LIAAEDEIHFA...........AASSPPFLHPRFIMD.......................................
m86008        ...ENPTPSLS...................................FFQDFLDHGNDIVG........SFDCDELRREVERLLE........................................
m31640        ..LPESSPSSYLFP.................................EHFDSIDPLMEFWN..SILSQPPDGSTFQNSQKYVINR.......................................
VQ7           HHPTADDDNHQLMMR............................QFSEFDRRTEKPPEIHNNISSSSSAPIKGSSGTTTTTTTTVDP............WICPTDSNN.................
m143752       .QSDVTTSFMN.....................................FFNERSERQQNFVTDVNSSNTWDEMLRIEYPGSTHIQ.......................................
VQ5           WFNGSTQEMHG....................................ASSLQSTRVDYEYP...LPLTPNFTFSSMTQHEVFGSDLDS............IGPDEDLEFSYEIN..........
m74628        ....VCDG..................................................................DAIQKPGFIIE.......................................
VQ28          ..EYSGNSS.........................................YDESLDHMDYY....QDFHRETATLEEFMMRDLDL.......................................
consensus>50  ............................................................................................................................

m24374        ............................................................................................................................
m30372        ............................................................................................................................
m77846        ............................................................................................................................
m70147        ............................................................................................................................
VQ31          ............................................................................................................................
AT5G084802    ............................................................................................................................
VQ11          ............................................................................................................................
VQ4           ............................................................................................................................
AT1G282802    ............................................................................................................................
VQ13          ............................................................................................................................
VQ19          ............................................................................................................................
VQ33          ............................................................................................................................
VQ3           ......FDS...................................................................................................................
VQ2           DMGFGQLDSVGRPGREPHPLPPDVSNTLYVEGLPSNCSRREVSHIFRPFVGYREVRLVTQDSKHRSGDPTVLCFVDFENSACAATALSALQDYRMDEDEPDSKILRLQFFRNPGPRPGQRGGRR
VQ8           ............................................................................................................................
VQ21          ....HIWDQD..................................................................................................................
m122290       ............................................................................................................................
m27654        ............................................................................................................................
m185931       ............................................................................................................................
m80937        ............................................................................................................................
VQ17          ............................................................................................................................
AT3G580001    ............................................................................................................................
VQ18          ............................................................................................................................
VQ26          ............................................................................................................................
VQ16          ............................................................................................................................
VQ23          ............................................................................................................................
VQ12          ...................RW.......................................................................................................
VQ29          ...................RW.......................................................................................................
AT5G467801    GFFFPL..........LSPLWKNQ....................................................................................................
VQ32          GFFFPL..........LSPLWKNQ....................................................................................................
VQ14          GLMFPLSP..SGFFPMPSPRWNDY....................................................................................................
m23084        GVMFPLQSPTSARFRLPSPRWRDSDMSFTFPDPDRRL.......................................................................................
VQ9           .TTVPL..........PSPKYKGH....................................................................................................
m112252       ............................................................................................................................
m2404         ............................................................................................................................
m127723       ............................................................................................................................
m1009         ............................................................................................................................
m109503       ............................................................................................................................
AtJAV1        ............................................................................................................................
VQ27          ............................................................................................................................
VQ30          ............................................................................................................................
VQ34          ............................................................................................................................
VQ15          ............................................................................................................................
VQ24          ............................................................................................................................
VQ10          ............................................................................................................................
VQ1           ............................................................................................................................
TcJAV3        ............................................................................................................................
m50381        ............................................................................................................................
m88532        ............................................................................................................................
VQ20          ............................................................................................................................
m104673       ............................................................................................................................
m70872        ............................................................................................................................
m86008        ............................................................................................................................
m31640        ............................................................................................................................
VQ7           ............................................................................................................................
m143752       ............................................................................................................................
VQ5           ............................................................................................................................
m74628        ............................................................................................................................
VQ28          ............................................................................................................................
consensus>50  ............................................................................................................................
```
