# Supplementary material for: TcJAV3–TcWRKY26 Cascade Is a Missing Link in the Jasmonate-Activated Expression of Taxol Biosynthesis Gene DBAT in Taxus chinensis
Source: Int J Mol Sci. 2022 Oct 29;23(21):13194. doi: 10.3390/ijms232113194 (PMC9656678; doi:10.3390/ijms232113194)
Supplement: Supplementary file 1 [file ijms-23-13194-s001.zip › Figure S5.pdf]

```
TcJAV3   ATGGAGTTTG---TGTCGCAGTCACACTTGGAAGACAGTCGCG-----TAATTTC   47
AtJAV1   ATGGCTAACCCCAACGAGTGGTCTCAGTTCTACAACAATAACCAAACCTTCTTCA   55

TcJAV3   CAATGTCTGGAACGGTTTCAGTTCTCCTCTGGAATTGGATTCCTCTGCTTCTTCC  102
AtJAV1   CAACTTCTACCACCGCTTCAAC-CGCTGTTACAACCA-CCACTG-----------   97

TcJAV3   TCCTCCTTGCGATTCCCCACTCAAAATCGTAGCTTGGAGACGTGGGTGCAGCTG-  156
AtJAV1   -----CCGGTGATACCACCTCCATAGACTCTCGTCTGAGTCCAGAAACTGGCCGT  147

TcJAV3   -------AAGAACCATTTGCAGGCGGATCAGAGCAAGGCCAACGGTGCCGTGAAC  204
AtJAV1   GTAACCAAGCCAAC--ACGTAGAAGGTCCAGAGCCTCACGTAGAACACCAACAAC  200

TcJAV3   TCTGAAGCGACGGCGGTGACGACGAAGGCGCTTCCGGCGCAGGG--CGGCCATTC  257
AtJAV1   GCTTC-TCAACACCG-ACACTTCCAA----CTTCCGTGCCATGGTTCAGCAATAC  249

TcJAV3   TTTTGTGGGCGGAGCAGCGACTGGTAAA--GTGGGGAAGAAACGATCGAGAGCTT  310
AtJAV1   ACTGGCGGTC-CATCCGCTATGGCTTTCGGGTCCGGTA-ATACTA-CTTCTGCTT  301

TcJAV3   CGCGGAGGGCGCCGACCACGCTTCTGAGCACTGATACTTCCAATTTCAGGGCCAT  365
AtJAV1   TTAGCCTCACTTCATCGTCGGATCCATCAGCTGGATCTTCTCAA-CAAGCTCCTT  355

TcJAV3   GGTGCAGCAATTCACAGGCATCCCCGAGTCGCCCTACGCCTCGCAGGGCAGATCC  420
AtJAV1   GGCAATATAATTTCCAGCC--TCACGCGCCGCTTCAGCCACCGCAACG----GCC  404

TcJAV3   TTGTACCGTTTTACTCCTCCGCAGCATAACTACTACTCCTCGTTTCTGTTCCAGG  475
AtJAV1   TTACAT-GTTTT-CT-TTGAACAACGTGA-----ATCCCGTGGTT-GGATATAGT  450

TcJAV3   ATCGCATGAATACGCCTCCATTTTTCCTTCCGAGGGTTCCTCCTCCTCCCCTCAA  530
AtJAV1   A--ACATGAACAACCCT--AA-------TACGATGGTTTCCGGT--------GTG  486

TcJAV3   TCCTTCTGCGGCGGAGGATTTTGGACTGAACCCTGTGTTTTCCGGCCCTCTAATG  585
AtJAV1   T-TTGGGACGGTGGATGGTTCTGG--TGGAGGAGGTTCTGCTCCGTCGTCAAAGG  538

TcJAV3   TTGCCTCATTTGGGGATTTCTG--ATAGCTTGATCAAATGA                624
AtJAV1   AGGCTACGAATAGTAATTCTTCTTCTTCGAGGTTGCAATGA                579
```
