# Supplementary figures and images for: TcJAV3–TcWRKY26 Cascade Is a Missing Link in the Jasmonate-Activated Expression of Taxol Biosynthesis Gene DBAT in Taxus chinensis
Source: Int J Mol Sci. 2022 Oct 29;23(21):13194. doi: 10.3390/ijms232113194 (PMC9656678; doi:10.3390/ijms232113194)

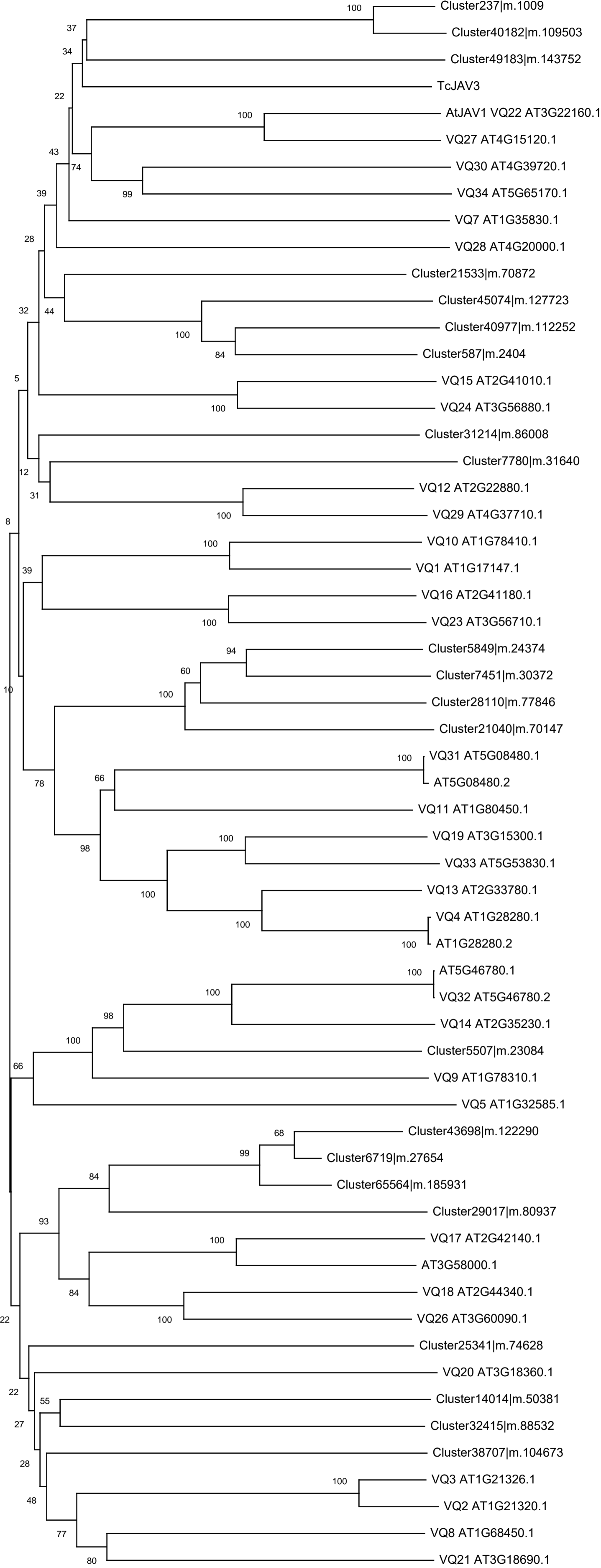

0.10

Supplement: Supplementary file 1 [file ijms-23-13194-s001.zip › Figure S3.pdf]

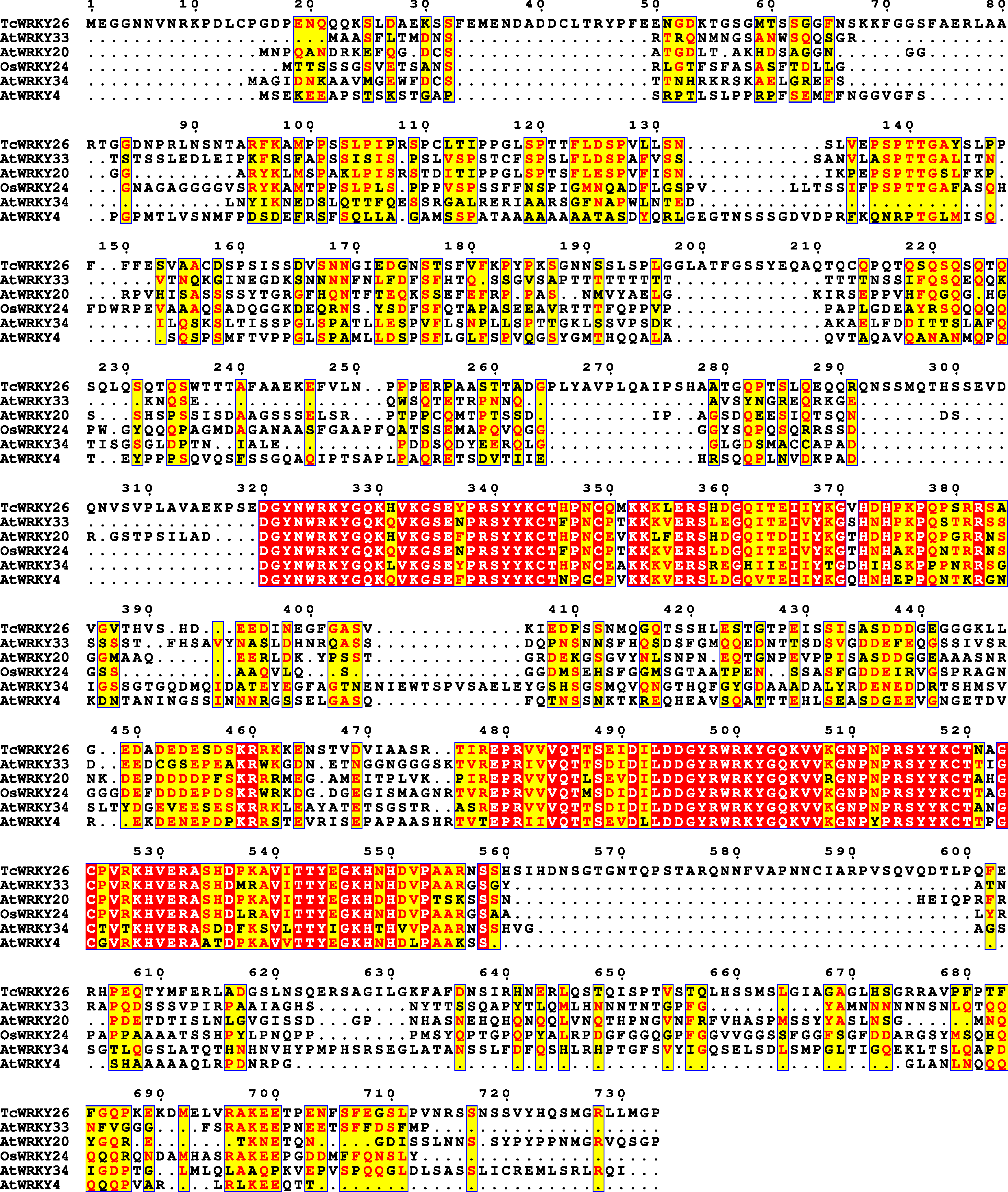

Supplement: Supplementary file 1 [file ijms-23-13194-s001.zip › Figure S4.tif]

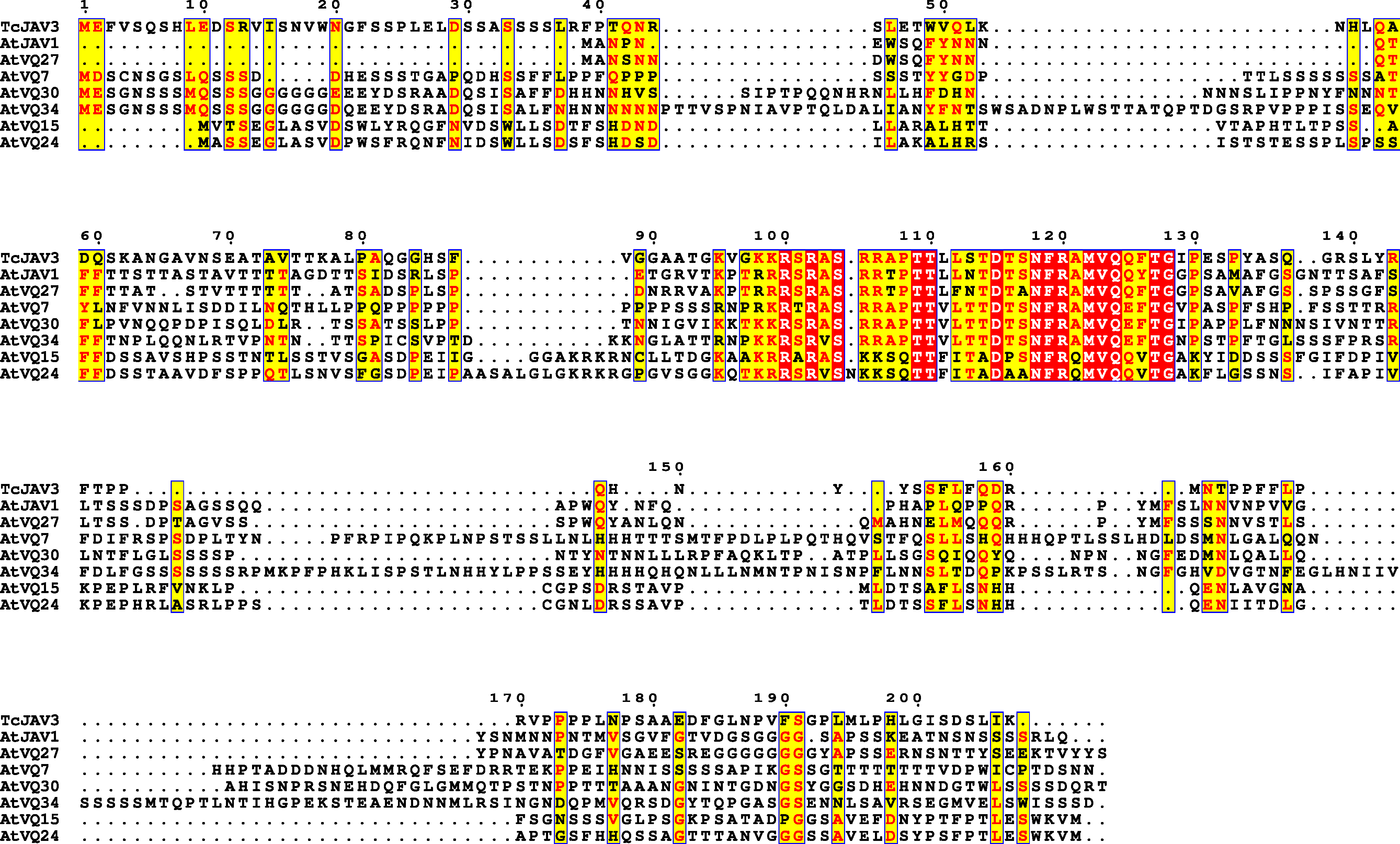

Supplement: Supplementary file 1 [file ijms-23-13194-s001.zip › Figures S1.tif]
